# Oxalic Acid Has an Additional, Detoxifying Function in *Sclerotinia sclerotiorum* Pathogenesis

**DOI:** 10.1371/journal.pone.0072292

**Published:** 2013-08-12

**Authors:** Annerose Heller, Tanja Witt-Geiges

**Affiliations:** Institute of Botany (210), University of Hohenheim, Stuttgart, Germany; Soonchunhyang University, Republic of Korea

## Abstract

The mechanism of the diseases caused by the necrotroph plant pathogen *Sclerotinia sclerotiorum* is not well understood. To investigate the role of oxalic acid during infection high resolution, light-, scanning-, transmission electron microscopy and various histochemical staining methods were used. Our inoculation method allowed us to follow degradation of host plant tissue around single hyphae and to observe the reaction of host cells in direct contact with single invading hyphae. After penetration the outer epidermal cell wall matrix appeared degraded around subcuticular hyphae (12-24 hpi). Calcium oxalate crystals were detected in advanced (36-48 hpi) and late (72 hpi) infection stages, but not in early stages. In early infection stages, surprisingly, no toxic effect of oxalic acid eventually secreted by *S. sclerotiorum* was observed. As oxalic acid is a common metabolite in plants, we propose that attacked host cells are able to metabolize oxalic acid in the early infection stage and translocate it to their vacuoles where it is stored as calcium oxalate. The effects, observed on healthy tissue upon external application of oxalic acid to non-infected, living tissue and cell wall degradation of dead host cells starting at the inner side of the walls support this idea. The results indicate that oxalic acid concentrations in the early stage of infection stay below the toxic level. In plant and fungi oxalic acid/calcium oxalate plays an important role in calcium regulation. Oxalic acid likely could quench calcium ions released during cell wall breakdown to protect growing hyphae from toxic calcium concentrations in the infection area. As calcium antimonate-precipitates were found in vesicles of young hyphae, we propose that calcium is translocated to the older parts of hyphae and detoxified by building non-toxic, stable oxalate crystals. We propose an infection model where oxalic acid plays a detoxifying role in late infection stages.

## Introduction

*Sclerotinia sclerotiorum* is a devastating fungal pathogen causing white mould of many plant species with enormous losses in a variety of economically important crops including sunflower [1]. The disease is difficult to control and up to now breeding for resistance has had limited success. Investigations on this necrotroph pathogen started as early as 1837 [2] and 1886 [3]. Since that time, many investigations have been performed, but still the interaction of the pathogen with its numerous hosts is not well understood. It is widely accepted that the key factor in pathogenesis of *S. sclerotiorum* is secretion of oxalic acid that act as an unspecific toxin [4-6], as well as numerous extracellular enzymes, especially polygalacturonases [7-9]. While *S. sclerotiorum* secretes several kinds of cell wall degrading enzymes that macerate the host tissue to provide nutrients for mycelial growth, oxalic acid seems to play multiple roles. Bateman [7] showed that oxalic acid acts synergistically with polygalacturonases, by lowering the pH and providing optimal conditions for the activity of the enzymes, and by chelating cell wall Ca^2+^ thereby providing polygalacturonases easy access to cell wall pectin. Oxalic acid interferes with defence mechanisms of host plants by inhibiting the activities of polyphenol oxidases [5] by suppressing the oxidative burst [10] and by manipulating the host redox environment [11]. It is an elicitor of programmed cell death in plants and responsible for induction of apoptotic-like features in the plant during disease development [12]. Also it causes wilting symptoms in sunflowers [13], and Guimaraes [14] showed that oxalate production by *S. sclerotiorum* deregulates guard cells during infection leading to foliar dehydration.

Oxalic acid/calcium oxalate is widespread in the plant, fungi, and animal kingdoms. In plants, functions are seen in calcium regulation, plant defense, and detoxification [15]. In fungi, it plays a role in pathogenesis, controls the availability of nutrients, regulates various aspects of soil chemistry, e.g. the level of Ca^2+^, detoxifies copper compounds [16] and degrade lignocellulose in wood-rotting fungi [17].

In *S. sclerotiorum* it is not known whether secretion of oxalic acid starts before or immediately after hyphal penetration of the host epidermis or later in the infection process. Most investigations dealing with oxalic acid production in *S. sclerotiorum* infected host plants did not include early infection stages, but stages with clearly visible lesions when oxalic acid levels were high in the killed tissue. Lumsden [18] performed light microscopical investigations of the initial infections stages of *S. sclerotiorum* on bean hypocotyl, but did not study the role of oxalic acid.

In order to resolve the role of oxalic acid in early infection stages we developed an inoculation method to follow the very fast and difficult to examine invasion process of *S. sclerotiorum* on sunflower hypocotyl with high resolution light-, scanning electron-, and transmission electron microscopy (TEM). We focused on individual infection cushions and single invading hyphae and the destruction process of host cell walls and other tissue caused by exuded enzymes and oxalic acid, and also looked for host cell reactions. For tracing oxalic acid exudates histochemical staining of calcium oxalate was used. Staining with potassium pyroantimonate followed Ca^2+^ release in the degraded tissue. Precipitation of calcium oxalate by CaCl_2_ gave us information about the occurrence of oxalic acid in low concentrations in infected tissue. Also the ability of the host cells to translocate oxalic acid and the potential destructive effect of oxalic acid was investigated on non-infected tissue of sunflower hypocotyl. Our results bring new insights concerning the multiple roles of oxalic acid in the plant–pathogen interaction involving *S. sclerotiorum*. We postulate that oxalic acid is important in balancing calcium levels at the infection site to prevent toxic calcium concentrations from inhibiting growth *S. sclerotiorum*.

## Results

### The infection process in high resolution

**Figure 1:**
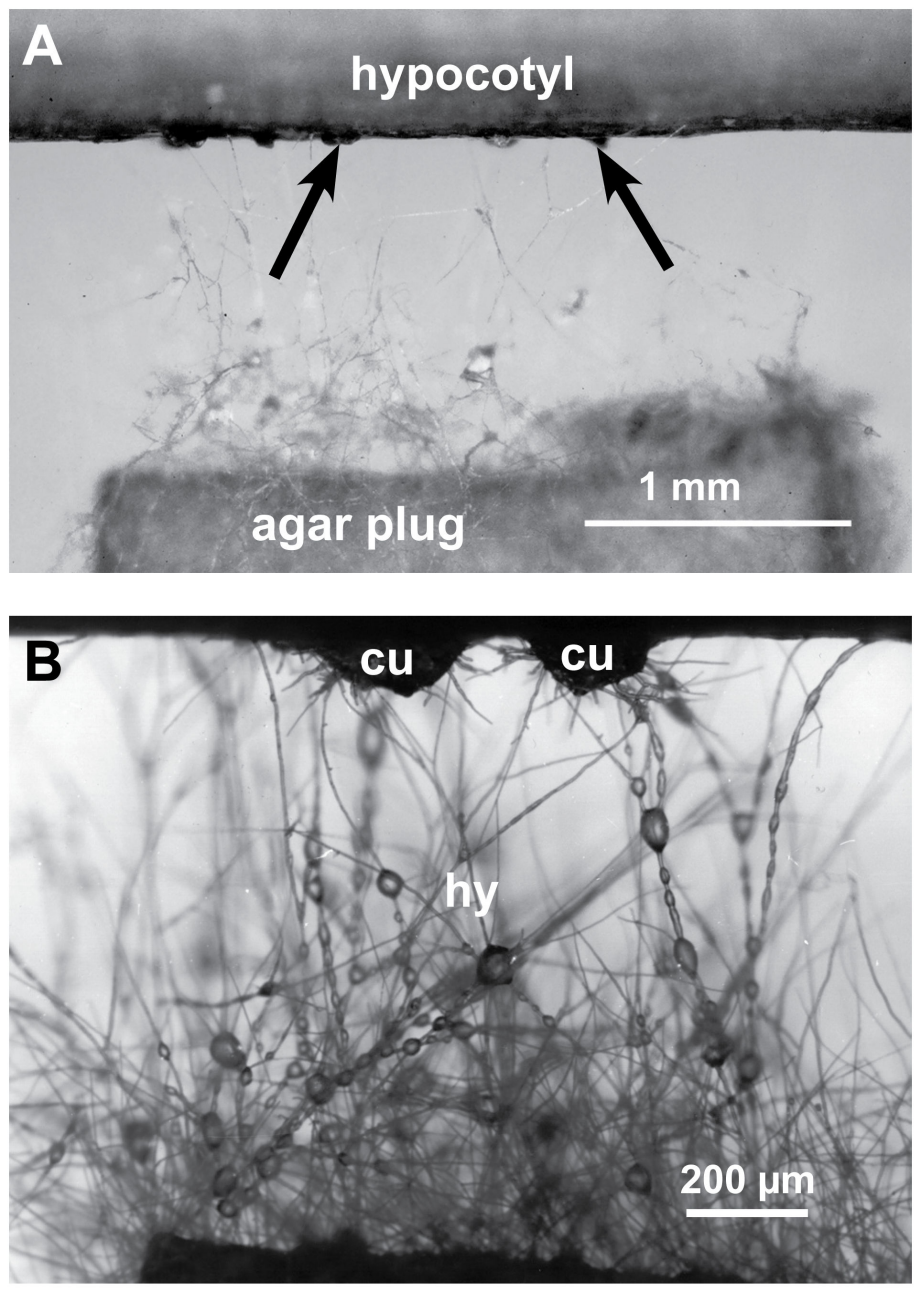
Inoculation method for following the early infection process. A piece of sunflower hypocotyl and an agar plug with actively growing mycelium of *S. sclerotiorum* were placed at a distance of about 1 mm in a Petri dish with wet filter paper. **A**: Aerial mycelium developed and when prospecting hyphae came in contact with the host surface dome-shaped infection cushions (arrows) developed at 12–24 hpi. **B**: Detail of Figure 1A; prospecting hyphae (hy) of the aerial mycelium with water droplets and two infection cushions (cu).

To investigate of the role of oxalic acid it is important to know the sequence of events in the *S. sclerotiorum* infection process. Previous published information often lacked sufficient detail. Also the extremely fast progress of the infection posed difficulties. We therefore, developed an inoculation method by placing agar plugs at a small distance to the host tissue, which allowed us to follow the infection process from the very beginning and to investigate it with various microscopic high-resolution methods (Figure 1A). We found that development of prospecting hyphae and infection cushions (Figure 1B) were similar to mycelial infection of sunflower stems under natural conditions.

**Figure 2:**
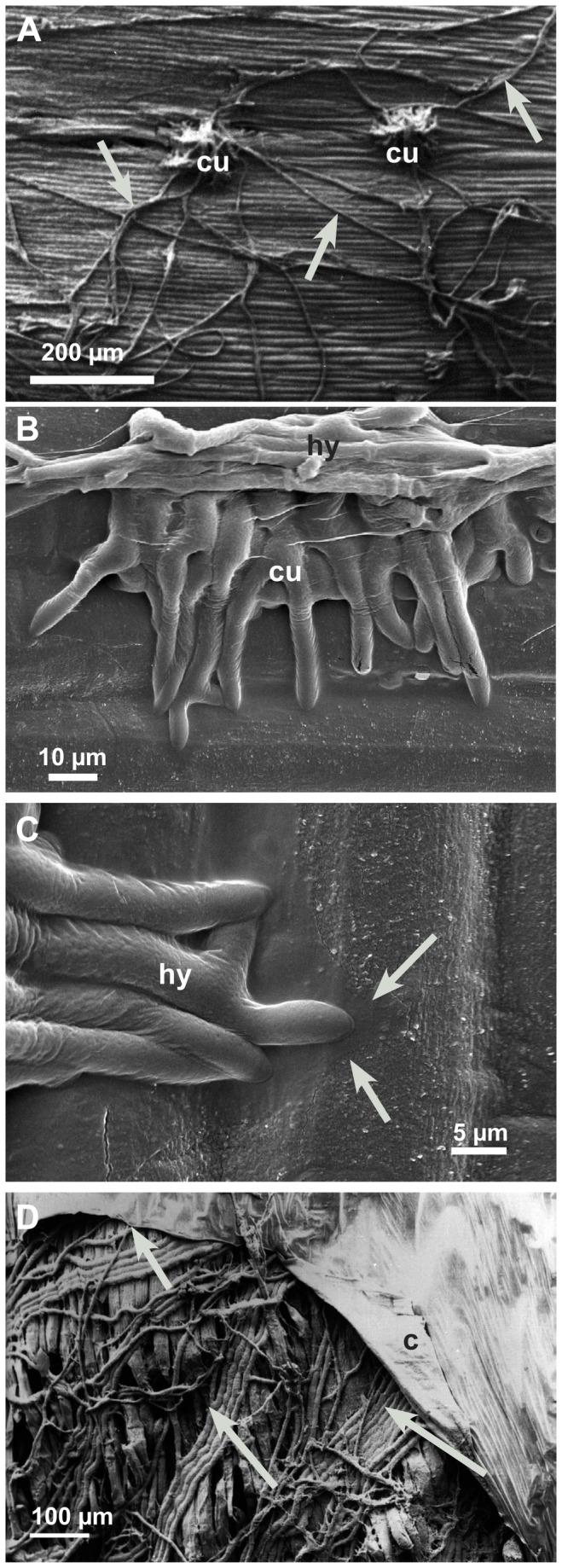
Development of early infection structures (12-24 hpi). Infection cushions, running hyphae on the epidermis of hypocotyls, and subcuticular hyphae investigated by scanning electron microscopy. **A**: Dome-shaped infection cushion (cu) and running hyphae (arrows); conventional SEM. **B**: Young infection cushion (cu) overgrown by running hyphae (hy); LTSEM. **C**: Detail of Figure 2B. Hyphae (hy) of the infection cushion attached to the cuticle of the epidermis. Hyphal exudates (arrows) covering wax crystals of the cuticle in LTSEM. **D**: After penetration, when the cuticle (c) is detached from the epidermal layer the parallel growing subcuticular hyphae (arrows) become visible in conventional SEM.

**Figure 3:**
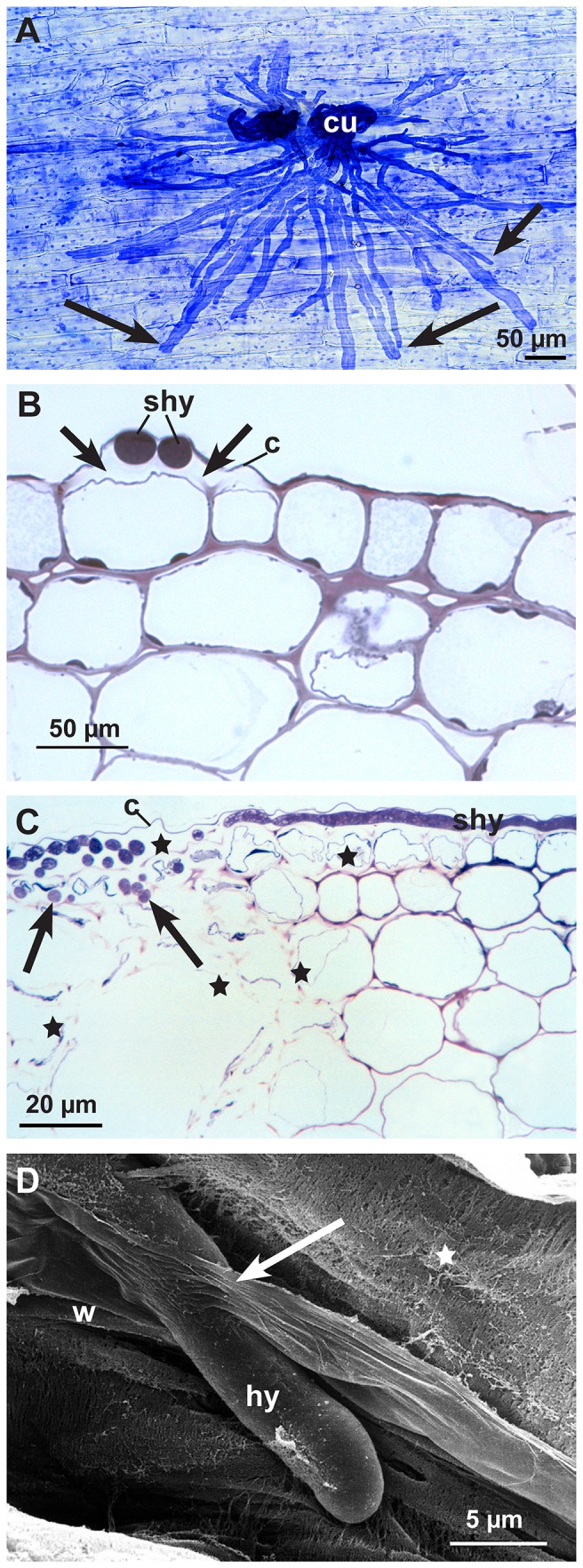
Infection process of the early (12-24 hpi) and advanced infection stages (36-48 hpi). Subcuticular hyphae and hyphal growth starting in epidermal and adjacent cortical parenchyma cells. **A**: Subcuticular hyphae (arrows) spreading fan-like from two infection cushions (cu). Light micrograph of an epidermal strip stained with Coomassie blue 12-24 hpi. **B**: Cross section of subcuticular hyphae (shy) near hyphal tips in the abaxial cell wall. Under the cuticle (c) around hyphae the loss of contrast and the widening of the cell wall indicating cell wall degradation (arrows). Light micrograph stained with toluidine blue 12-24 hpi. **C**: Cross section of infected hypocotyl with subcuticular hyphae (shy) under the cuticle (c) and hyphae growing deeper into the host tissue (arrows). Cell walls and cytoplasm of the epidermal cells are completely destroyed as well as parts of the cortical parenchyma (stars). Light micrograph stained with toluidine blue 36-48 hpi. **D**: Scanning electron micrograph, 36-48 hpi. In a cortical parenchyma cell the fibrillar (stars) and lamellar (arrow) structure of the degrading cell wall (w) becomes visible around an invading hyphae (hy) by conventional SEM.

**Figure 4:**
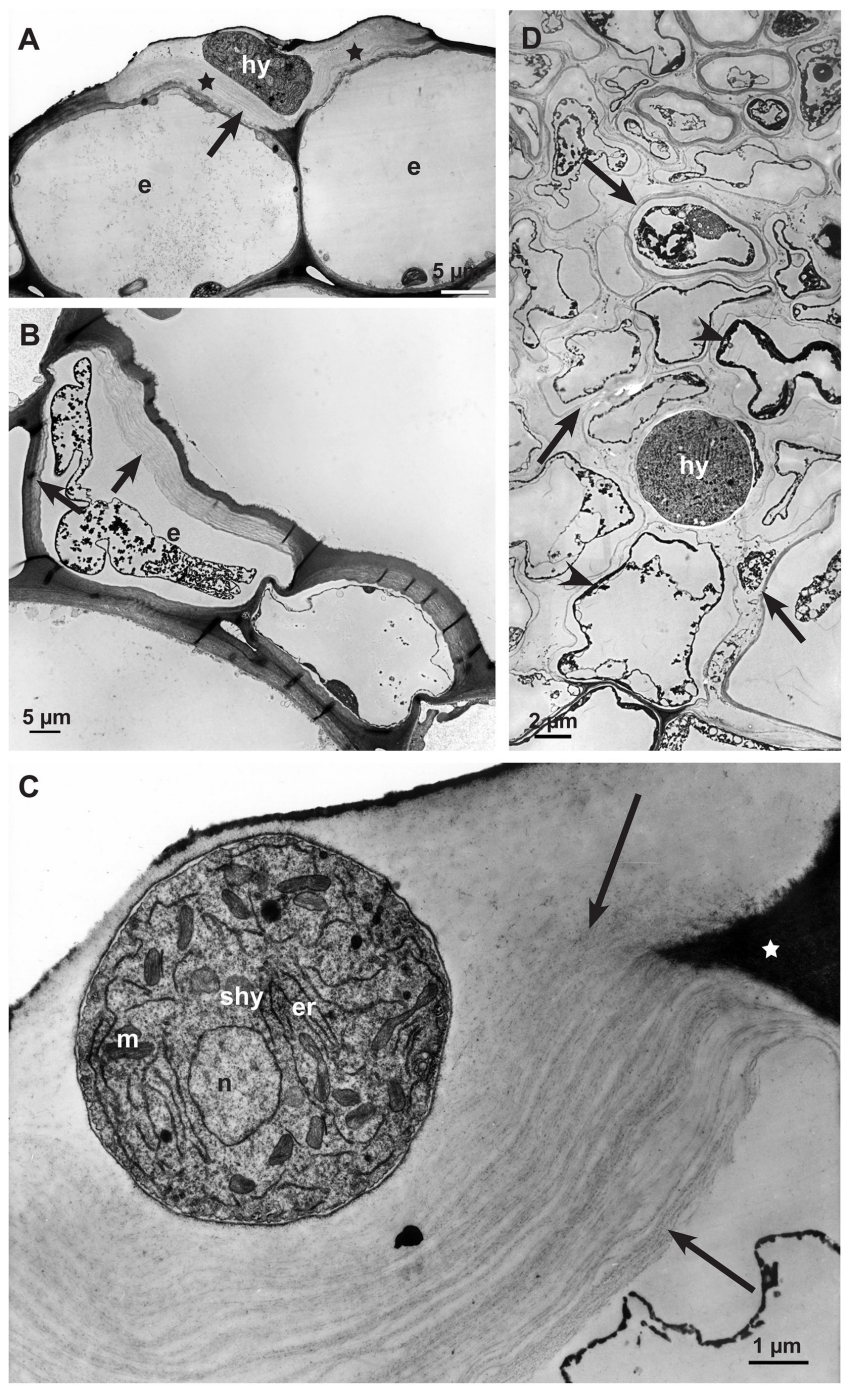
Degradation process of the cell wall matrix in early (12-24 hpi) and advanced (36-48 hpi) infection stages. Transmission electron micrographs of cross sections of sunflower hypocotyls: **A**: The abaxial epidermal cell wall around a subcuticular hypha (shy) 12-24 hpi showing degraded cell wall matrix (stars). Cytoplasm of the epidermal cells (e) still intact. **B**: Destruction process of cell wall in dead host cells starting from the inner side of the cell wall (arrows) of an epidermal cell (e) 12-24 hpi. **C**: Detail of a degraded abaxial epidermal cell wall around a subcuticular hypha (shy); non-degraded part of the plant cell wall (star), cell wall matrix degraded, residues of cellulose layers (arrows), nucleus (n), endoplasmic reticulum (er) and mitochondrium (m) of the fungal cell. **D**: During the advanced infection stage (36-48 hpi) a single hypha (hy) in a large area of the necrotic cortical parenchyma. Only residues of thin cell wall layers (arrows) and dark staining residues of cytoplasm are left (arrowheads).

Prospecting hyphae of *S. sclerotiorum* started to grow 10-16h after the agar plug was placed close to the hypocotyl. After 12-24 hpi, the first infection cushions formed on the hypocotyl surfaces (Figure 1A,B). The following three infection stages were chosen for microscopy studies: 12-24 hpi (early), 36-48 hpi (advanced), and 72 hpi (late). Scanning electron microscopy (SEM) and Coomassie blue-stained strips of epidermal layers showed the development of *S. sclerotiorum* at the infection sites on the hypocotyl (Figure 2A and Figure 3A). In early infection stages (12-24 hpi), shortly after prospecting hyphae had contact with the epidermal layer, dome-shaped infection cushions composed of many short hyphae (appressorial hyphae) developed quickly (Figure 2A, B). From infection cushions so called running hyphae appeared and grew over the epidermal surface (Figure 2A). Some hyphae gave rise to new infection cushions at a distance. Hyphae of the infection cushion were fixed tightly to the plant surface by exuded material, which was also detectable around hyphae on the epidermis using low temperature scanning electron microscopy (LTSEM) (Figure 2C). Both, appressorial hyphae of infection cushions and running hyphae were able to directly penetrate the cuticle of the epidermal layer (not shown). After penetration, hyphae were growing under the cuticle, in the abaxial cell wall of the epidermal cells (Figures 2D, 3A, 3B), hence called subcuticular hyphae (shy). They developed fan-like from the infection cushions and many of them were oriented parallel building an infection front (Figures 2D and 3A). Also the first signs of host tissue degradation became visible around these subcuticular hyphae in the early infection stage. The abaxial host cell wall matrix disappeared, whereas the cytoplasm was still intact (Figure 3B). In conventional SEM and TEM destroyed cell walls with loose cellulose layers became visible (Figures 3D and 4A, B, C). While the infection was progressing, the cell wall matrix of host cells disappeared not only around hyphae, but also at a distance. The degeneration process distant from hyphae in already killed host cells started from the inner side of the host cell wall and proceeded to the outside (Figure 4B). In the advanced and late stages, 36-48 hpi and 72 hpi, when the epidermis was already destroyed, hyphae of variable diameters colonized the cortical parenchyma growing inter- and intracellularly (Figures 3C, 4D). The host tissue degraded gradually up to a distance of three to five cell layers from the hyphae (Figure 3C). In the late stage of infection typical necrotic lesions became visible. In the area of the necrotic lesions cytoplasm and cell walls appeared degraded and in the end only parts of the middle lamella, cellulose layers of the walls, and remnants of cytoplasm were visible (Figure 4D). Extensive colonization of the tissue by hyphae started, when host cells were already dead. In the late and sometimes in the advanced infection stages, hyphae of the infection cushion and subcuticular hyphae around them became senescent and died (not shown).

**Figure 5:**
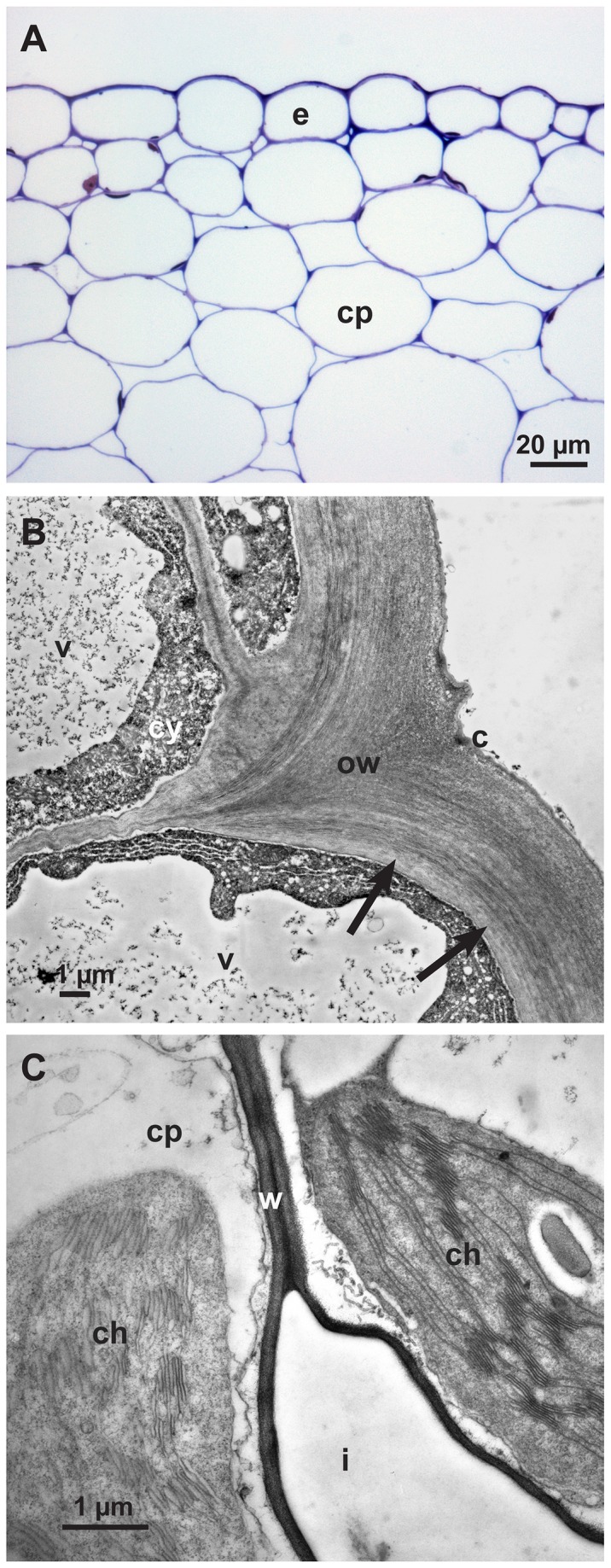
Non-infected tissues of sunflower hypocotyl (control). **A**: Light micrograph of a cross section of a sunflower epidermis (e) and cortical parenchyma (cp) stained with toluidine blue. **B**: Transmission electron micrograph showing detail of an abaxial epidermal cell wall (ow) with brighter inner layer (arrows), cuticle (c), cytoplasm (cy) and vacuole (v). **C**: Transmission electron micrograph showing detail of cortical parenchyma cells (cp), cell wall (w), chloroplast (ch) and intercellular space (i).

Tissues of non-infected hypocotyls showed living cells surrounded by non-degraded cell walls evenly stained in light microscopy as expected (Figure 5A). In conventional TEM the cell walls exhibited the typical structure and contrast of healthy plant cell walls. The adaxial cell walls of epidermal cells were often a little brighter in contrast than the abaxial walls under the cuticle (Figure 5B). The thin cell walls of the cortical parenchyma were even in contrast (Figure 5C).

**Figure 6:**
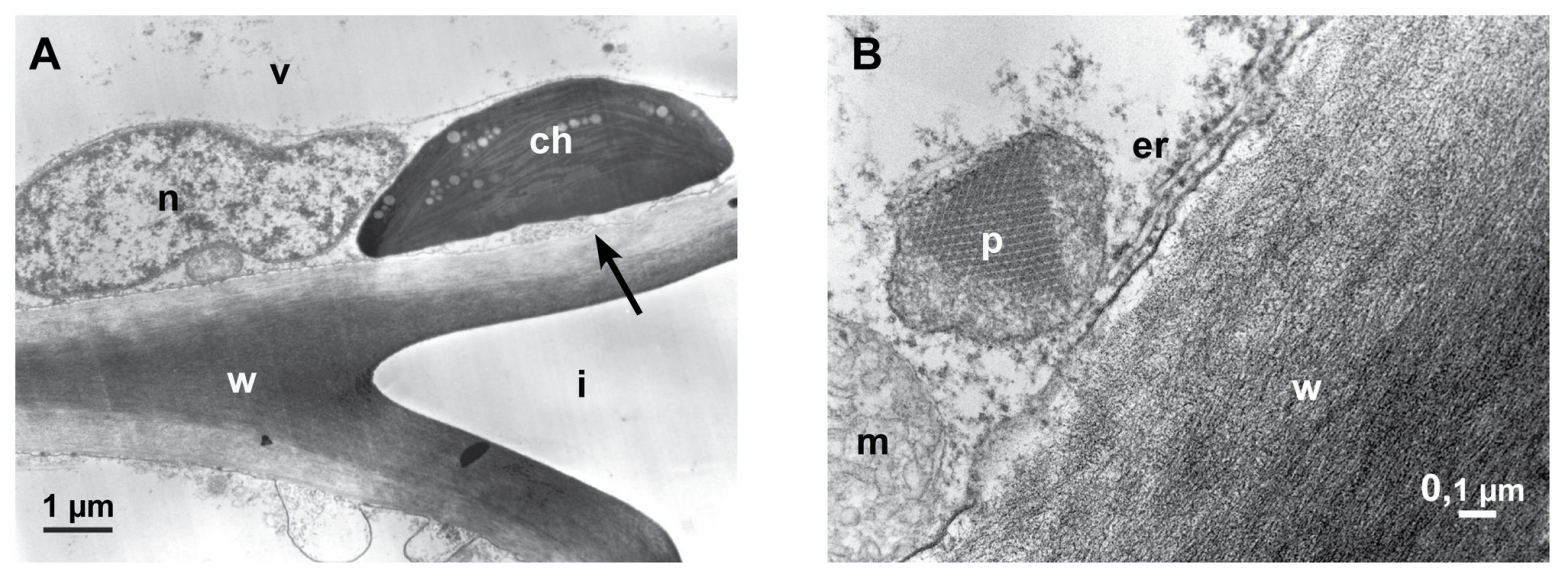
Reaction of the host cells. Transmission electron micrographs (12-24 hpi). **A**: A chloroplast (ch) changing contrast, the matrix appear electron dense. The brightening of the inner cell wall layer is probably the beginning of the degradation of the cell wall (arrow), nucleus (n), vacuole (v), intercellular space (i) and cell wall (w). **B**: A cortical parenchyma cell with peroxisome (p) containing protein-crystal (probably catalase), mitochondrium (m), endoplasmic reticulum (er) and cell wall (w).

Besides a beginning brightening and degradation of the cell walls, cell organelles were affected in early stages of infection (Figure 6A). In cells near infection sites the chloroplasts changed contrast, the intrathylakoid space and the matrix of the chloroplasts became electron dense (Figure 6A), and peroxisomes containing crystals (probably high concentrations of catalase) were conspicuous (Figure 6B).

### The secretion of oxalic acid in the infection process of *S. sclerotiorum*

**Figure 7:**
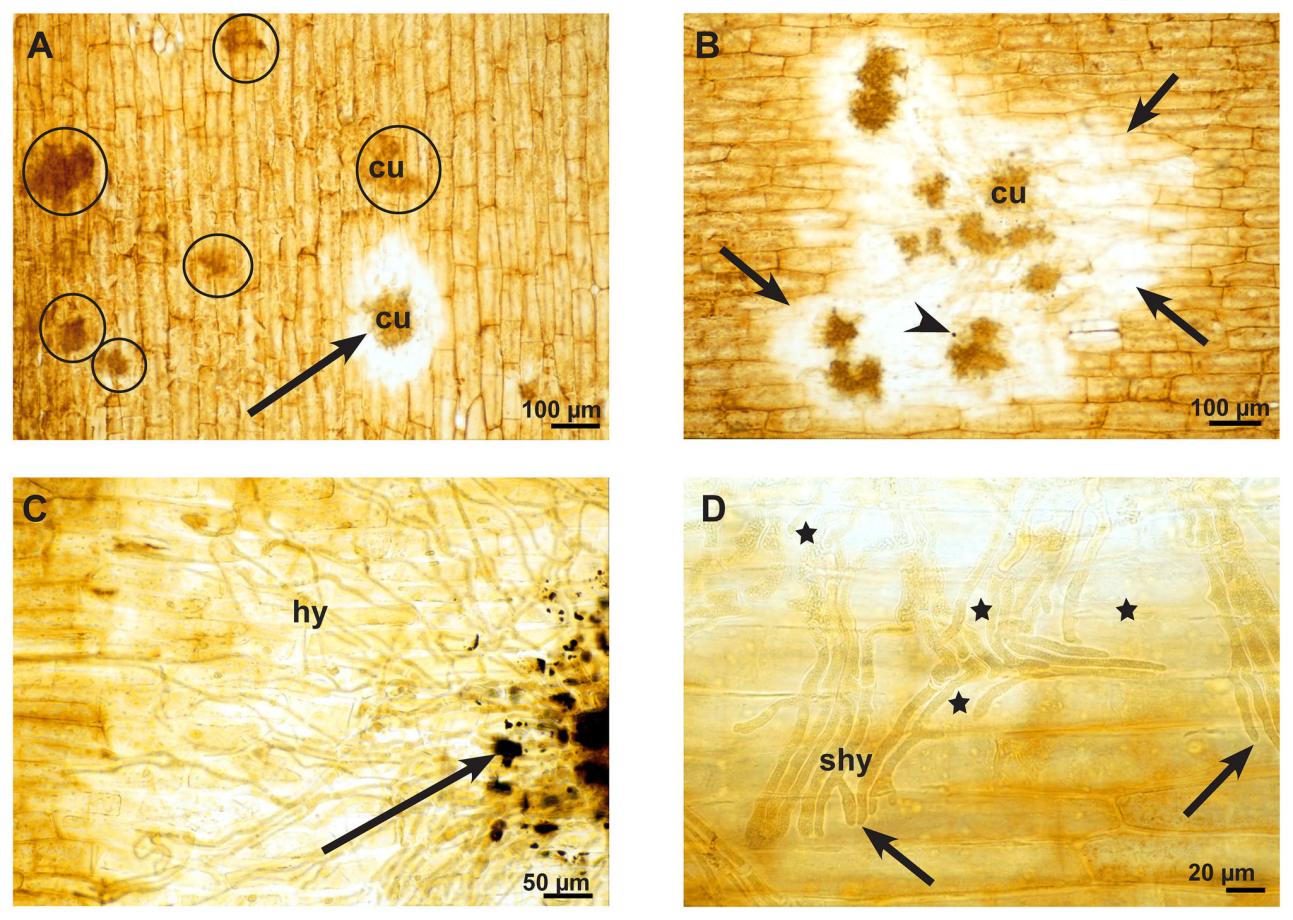
Secretion of oxalic acid in the infection process of *S. sclerotiorum*. Light micrographs of the early, advanced, and late stages of infection after histochemical staining for calcium oxalate of fresh epidermal strips that resulted in a yellow-brownish colour of tissue and a brown-black colour of the compact oxalate crystals. **A**: At the early infections stage (12-24 hpi) the epidermal layer around one infections cushion (cu) is bleached (arrow), while most of the infection cushions (circles) do not show any influence on the epidermal layer. **B**: In the advanced stage (36-48 hpi) there is bleaching of the epidermal layer (arrows) around all infection cushions (cu) and only a single dark stained particle of oxalate was visible (arrowhead). **C**: At the late stage (72 hpi) there is not only bleaching, but calcium oxalate crystals accumulate around the infection cushions (arrow). **D**: Detail of a late stage at the infection front. The bleaching effect (stars) did not become visible at the hyphal tips (arrows), but along the older parts of the subcuticular hypha (shy).

**Figure 8:**
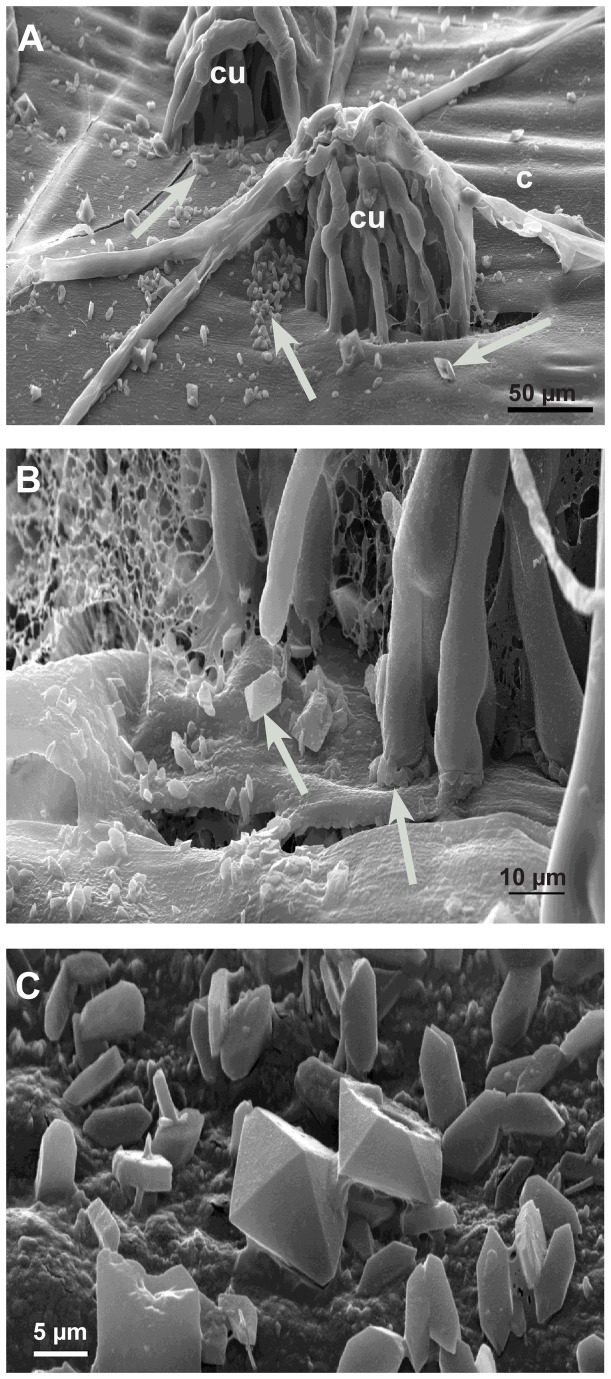
Calcium oxalate accumulation in the late infection stages (72 hpi) by LTSEM. Scanning electron micrographs: **A**: On the detached cuticle (c) calcium oxalate crystals appear (arrows) around the infections cushions (cu) **B**: Detail of Figure 8A showing the accumulation of crystals (arrows). **C**: Detail showing the variation of calcium oxalate crystals.

**Figure 9:**
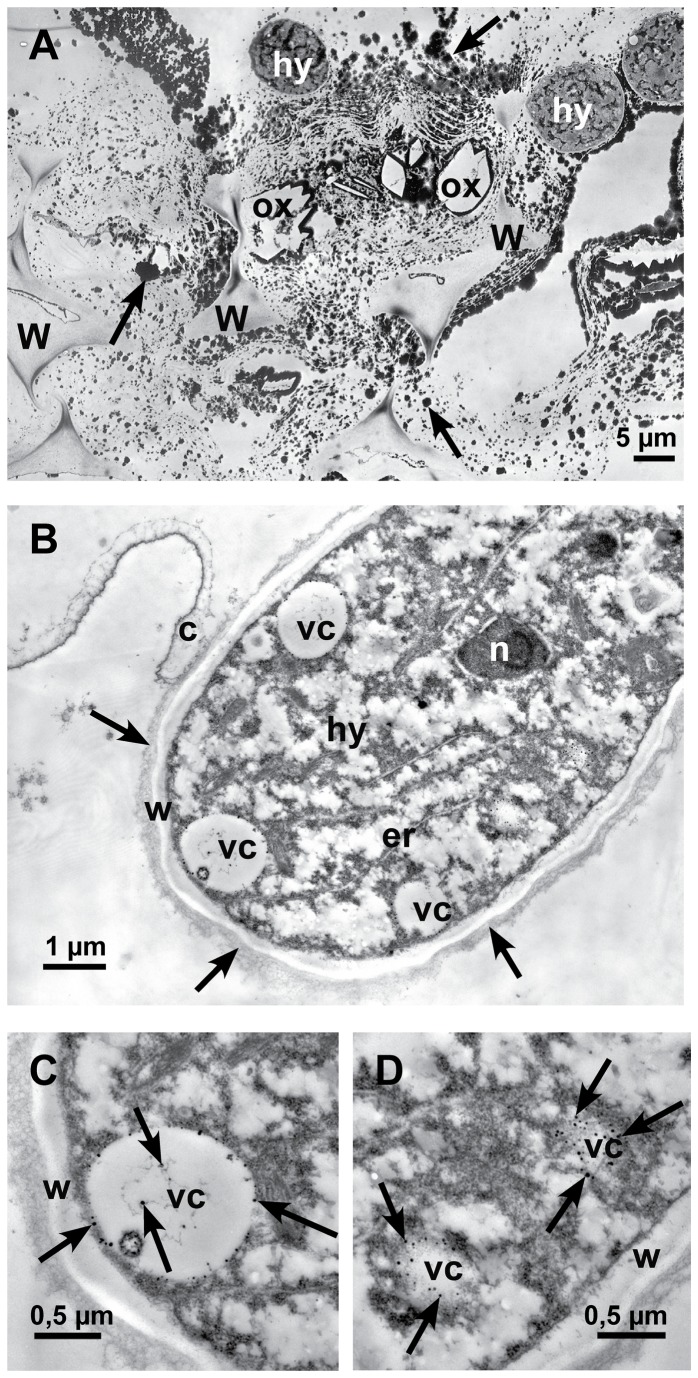
Localization of calcium by potassium pyroantimonate precipitation. Transmission electron micrographs of cross sections of sunflower hypocotyls. **A**: In the late infection stage (72 hpi), near an infection cushion, dark staining precipitates (arrows) are detectable all over in the destroyed tissue, around hyphae (hy) and oxalate crystals (ox) (crystals itself were lost during sectioning). Only residues of cell walls (w) are left. **B**: In the early infection stage 12-24 hpi, the longitudinal section of a hyphal tip (hy) that just penetrated the cuticle (c) is showing vesicles (vc) with small dark stained calcium precipitates. Around the cell wall of the hypha an electron dense, fibrous matrix is visible (arrows), nucleus (n), endoplasmic reticulum (er), cell wall (w) and vesicles (vc) with calcium precipitates. **C**: Detail of Figure 9B: Cross section of a vesicle (vc) with dark stained calcium precipitates (arrows) and cell wall (w). **D**: Detail of Figure 9B: Tangential section of vesicles (vc) with dark precipitates of calcium (arrows) and cell wall (w).

Oxalic acid is a low molecular acid that cannot be stained histochemically. However, oxalic acid reacts easily with calcium to produce calcium oxalate (thermodynamic solubility product, K_sp_,_th_ at 25°C = 2.32x 10^-9^ ,mol^2^ L^-2^) which can be stained. Therefore, if oxalic acid is secreted in plant tissue that contains calcium, calcium oxalate will precipitate easily and build recognizable oxalate crystals. Calcium oxalate as well as calcium carbonate and calcium phosphate are stained dark brown to black using a method described by Yasue [19]. According to Yasue, before staining of calcium oxalate, carbonate and phosphate salts need to be removed by a washing step with acetic acid. In this way infected and non-infected epidermal strips showed a general yellow brownish background colour. In the very early infection stage (12-24 hpi) the epidermal layer around some infection cushion did not change in colour, while others showed a clear halo around the infection cushions, where the general brownish colour had disappeared (Figure 7A). In the advanced stage (36-48 hpi), the host tissue around all infection cushions showed this bleaching effect (Figure 7B). Only in the late stage (72 hpi) did dark brown-black stained crystals of variable sizes accumulated around the infection cushions (Figure 7C). At that time, hyphae of the infections cushions and hyphae nearby were already dead and without function. Higher magnifications revealed that the bleaching of the host tissue did not start around the hyphal tips but at a distance from the tips along the hyphae (Figure 7D). Also in LTSEM, where crystals are not washed away by preparation, typical oxalate crystals of variable sizes and forms accumulated around infection cushions only at the late infection stages (72 hpi) (Figure 8A, B, C). Even in conventional TEM crystals accumulated in the destroyed host tissue under the infection cushions in the late infection stages (Figure 9A). Calcium oxalate crystals never appeared around running hyphae or infection cushions on the epidermal layer at early infection stages, and could also not be induced by the application of calcium chloride (not shown).

In the early infection stages, dark stained particles of calcium potassium pyroantimonate appeared only in vesicles of young hyphae (Figure 9B, C, D) after precipitation by potassium pyroantimonate. In contrast, in the late infection stages masses of dark staining calcium precipitates accumulated in the destroyed host tissue around hyphae, as well as oxalate crystals, and residues of cell walls (Figure 9A).

### The effect of oxalic acid on non-infected living and inactivated host tissue

**Figure 10:**
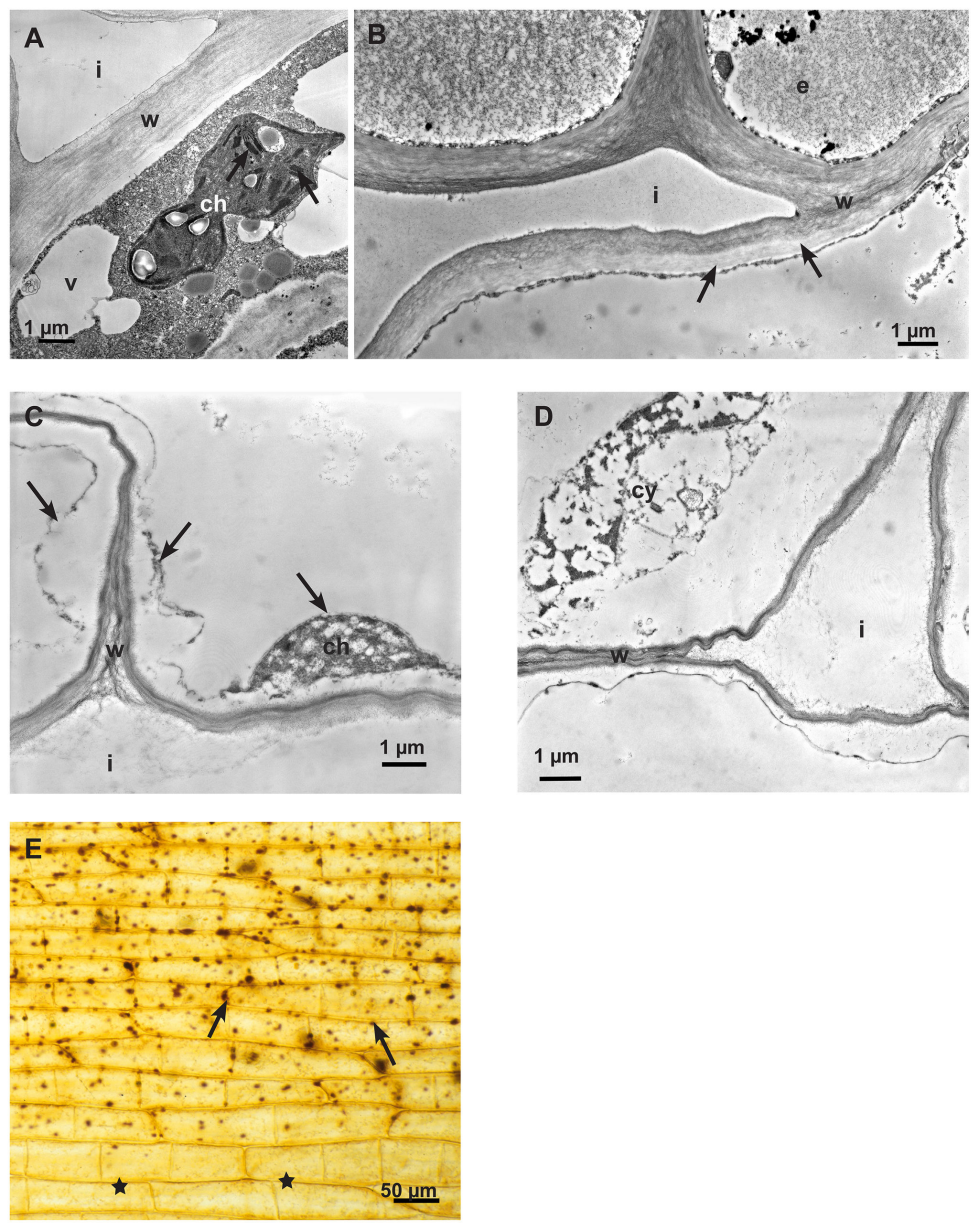
The Effect of oxalic acid on non-infected, living host tissue. Transmission electron micrographs of cross sections of sunflower hypocotyls. **A**: At a distance from the application of oxalic acid, one of the first effects was on chloroplasts. The intrathylakoid space changed contrast and became electron dense (arrows), chloroplast (ch), intercellular space (i), vacuole (v) and cell wall (w). **B**: Also in a distance from the application point, the cell walls lost contrast and brightened up from the inner side of the cell (arrows), epidermal cell (e), cell wall (w) and intercellular space (i). **C**: Detail of cortical parenchyma cells closer to the application point showing more severe effects: Cell walls (w) appeared degraded and the cytoplasm (arrows) severely damaged, residue of a chloroplast (ch) and intracellular space (i). **D**: Detail of cortical parenchyma cells close to the application point showing increased destruction effects on cytoplasm (cy) and cell walls (w) and intracellular space (i). **E**: Light micrograph of oxalic acid treated epidermal strip after histochemical staining of calcium oxalate (Method by Yasue). The effect of the oxalic acid becomes visible by brown staining particles (arrows), probably chloroplasts. Tissue without contact to oxalic acid (stars). There was no bleaching effect visible.

The failing histochemical detection of oxalic acid in the early infection stages was surprising because, if oxalic acid would act as a toxin and be a key factor of infection, a distinct oxalic acid secretion should be expected. To examine whether the host tissue was interfering, e.g. by metabolizing the fungal oxalic acid, the effect of drops of oxalic acid (5 mM) was examined on non-infected, living tissue of sunflower hypocotyls. As oxalic acid did not penetrate the cuticle, it was perforated with a fine needle under the drop. The first effect of oxalic acid was visible on chloroplasts. The intrathylakoid space became electron dense (Figure 10A) and a brightening of the inner cell wall layer also was detectable (Figure 10B). In the advanced states the cytoplasm appeared degraded and a progressing decomposition of cell walls was obvious (Figure 10C, D), however, oxalate crystals were not formed. When oxalic acid treated, living epidermal strips were stained for oxalate [19], calcium oxalate crystals did not appear, nor was any bleaching effect visible. Only a darker staining of chloroplasts showed the effect of oxalic acid (Figure 10E) compared to untreated parts of the tissue.

**Figure 11:**
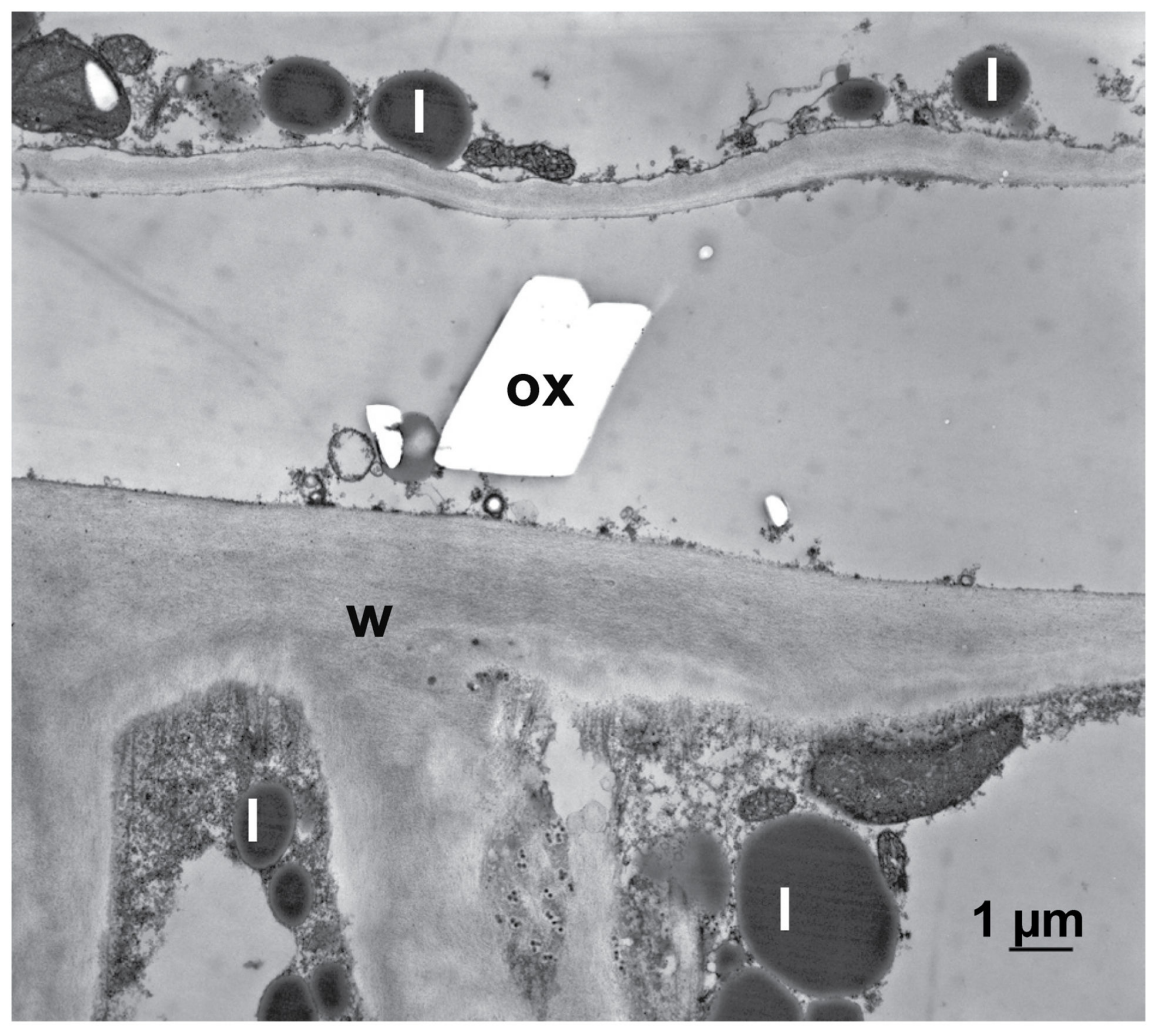
The Effect of oxalic acid on non-infected, inactivated host tissue. Transmission electron micrograph of a cross section of sunflower hypocotyl. After inactivating the tissue by glutaraldehyde fixation it was treated with oxalic acid and additionally with calcium chloride to precipitated calcium oxalate: In the inactivated tissue calcium oxalate crystals (ox) appeared. The accumulation of lipid bodies (l) is indicating the beginning of decomposition of the membranous system of the cell; cell wall (w).

Oxalate crystals only appeared when host tissue was inactivated by glutaraldehyde and then treated with oxalic acid and additionally with calcium chloride to precipitate oxalate (Figure 11). In contrast to this, no calcium oxalate crystals appeared when living, active host tissue was treated with calcium chloride after the application of oxalic acid (not shown). The effect of oxalic acid on cytoplasm and cell walls of inactivated tissue by glutaraldehyde was not severe compared to living tissue probably because of the protein fixation effect of glutaraldehyde, but the accumulation of lipid bodies indicated a beginning decomposition of the membranous system of the cytoplasm (Figure 11).

## Discussion

*Sclerotinia sclerotiorum* is a necrotrophic plant pathogen causing soft rots (white mold) in more than 400 plant species worldwide [1]. Not only biotrophs, but also necrotrophs establish a host parasite interaction [20,21]. Therefore, the very first infection stages seem to be of crucial importance for the interaction of *S. sclerotiorum* with sunflower. Most of previous investigations lacked observations of early infection stages and used inoculation techniques by which agar plugs with mycelium were placed directly on the host tissue resulting in massive invasion of hyphae without formation of infections cushions as observed under natural conditions. Williams [11] investigated the host redox environment from 8–18 hpi. In soybean hypocotyls the production of polygalacturonases and oxalic acid was investigated 12 hpi by Favaron [22]. Therefore, the timing is not comparable to our inoculation method with infection cushion development. Only Lumsden [18] showed a temporal progress of the infection in *S. sclerotiorum*-infected bean hypocotyl from 6 hours post inoculation to 7 days post inoculation comparable to our results in sunflower by using infested oat kernels for inoculation.

In sunflower cotyledons oxalic acid production increased significantly once 40% of leaf area had become necrotic [23]. It is well known that the severity of symptoms (lesions) is accompanied by high level of oxalic acid in the infected tissue. Many reports show that reduction of oxalic acid production is accompanied by reduction in pathogenicity [6,10,14,24,25]. Furthermore, mutants deficient in oxalic acid are non-pathogenic [4]. Transgenic plants transformed to express oxalate oxidase [26,27] or oxalate decarboxylase [28] were partially resistant to white mold or at least show reduced disease symptoms and a delayed colonization. The role of oxalic acid as a toxin for host plants in the infection process of *S. sclerotiorum* is emphasised in many publications, although oxalic acid accumulation is not always strongly related to pathogenicity [29]. There are moderately virulent and hypovirulent isolates which produce more oxalic acid than virulent isolates [30,31].

Oxalic acid is discussed as a key factor in the pathogenesis of *S. sclerotiorum*. It is believed that it acts as an unspecific toxin and supports cell wall break down by disintegrating the calcium-pectate complexes of cell walls, probably in a sequence followed by the activity of various cell wall degrading enzymes, especially polygalacturonases. A synergistic action of oxalic acid and polygalacturonases was shown by Bateman [7], however, only endopolygalacturonases have their optima below pH 6, while pectin lyases are more active above pH 6 [32]. As reviewed by Bolton [33] *S. sclerotiorum* produces a wide range of cell-wall-degrading enzymes during infection. This includes various polygalacturonases and proteases [21]. A sequential expression of the endopolygalacturonase-encoding genes was shown during pathogenesis and transcripts of the pg *1*-*3* genes started eight hours after the beginning of infection and reached a maximum 36 hpi [34]. *S. sclerotiorum*, like *Botrytis cinerea* can be regarded as a pectolytic fungus [32], as both prefer monocot species with high pectin content in their cell walls [33]. For *B. cinerea* it was shown that an endopolygalacturonase is required for full virulence [35]. In *S* *. rolfsii* highly virulent strains characteristically had rapid growth rates and produced large quantities of cell wall degrading enzymes and oxalic acid. In contrast, weakly virulent and less rapidly growing strains produced very low amounts of endo-polygalacturonase but showed no difference in oxalic acid production [36]. This indicates that oxalic acid may not be the decisive factor for the progression of the fungal hyphae in the plant tissue. During investigation of early infection stages by TEM it was obvious, that the first effect was degradation of the cell wall matrix consisting mostly of pectins, but also hemicelluloses and glycoproteins. Also the brightening effect around infection cushions on the epidermal strips occurring in the early infections stages seemed to be caused by the activity of cell wall-lytic enzymes since oxalic acid alone did not cause any brightening effect. While the effect of lytic enzymes on host cells walls was early visible in the current study, we did not observe oxalic acid/calcium oxalate in the early stages of infection of sunflower. Only in the advanced and late stages of infection, substantial amounts of calcium oxalate were found. This corroborates with results reported for *Brassica napus*, where oxalate crystals were not found until 6 days after inoculation [37]. Oxalic acid is interfering with calcium-mediated signalling and programmed cell death caused by a basic endopolygalacturonase produced early by *S. sclerotiorum* [38]. It is suppressing and manipulating the oxidative burst [10,11]. Therefore, it seems unlikely that oxalic acid is active at the early infection stage because it would delay the infection process of this necrotrophic pathogen.

*Per se* oxalic acid is not toxic to plant cells. It is a common metabolite in dicots and monocots. In *S. sclerotiorum* pathogenesis oxalic acid secretion probably starts in early infection stages as transcripts of oxaloacetate acetylhydrolase, the enzyme that catalyse the reaction from oxaloacetate to oxalic acid [39], were found already 8 hpi and during lesion formation [40]. As plants are able to deal with high concentrations of calcium as well as with oxalic acid and oxalate [41], we suppose that in the early infections stage, host cells interfere in the fungal oxalic acid secretion by metabolizing the oxalic acid and transferring it to host cell vacuoles. This idea is supported by several observations: 1. The appearance of peroxisomes with large protein crystals in host cells near invading hyphae is an indication of oxidative processes and might be involved in metabolization of oxalic acid. 2. We never found calcium oxalate around the invading subcuticular hyphae. 3. Also, oxalic acid was not detectable in living host cells near infections sites or in living non-infected tissue treated with oxalic acid and CaCl_2_. Only if the compartimentation of oxalic acid was abolished by inactivation, respectively killing the plant cells by glutaraldehyde fixation, oxalate crystals developed. 4. Furthermore, in dead host cells in infected and in non-infected tissue treated with oxalic acid we noticed that the cell wall degradation process started from the inner side (cytoplasmic side) of the cell wall and oxalic acid itself was able to degrade plant cell walls.

Oxalic acid and oxalate are closely linked to the calcium metabolism in cells [42]. In the apoplast a major part of calcium is bound to pectic acids of the matrix of the cell wall. In vacuoles high concentrations of calcium can be found in form of highly insoluble calcium oxalate crystals. For organisms in general, calcium is an important factor for metabolism, growth and cellular activity. Ca^2+^ acts as signal in many adaptation and developmental processes of plants, e.g. growth, abiotic stress, hormones, pathogens [43] and, therefore, cytoplasmic Ca^2+^-levels are low and highly regulated. For *Fusarium* *graminearum* it is known that the rate of hyphal extension and the amount of branching were both effected directly by the external Ca^2+^-concentrations [44]. In fungi, calcium gradients regulate tip growth and the uptake of ions from the external medium [45]. At high concentrations free calcium is a toxic cellular compound, because Ca^2+^ is building complexes with proteins, membranes, phosphate and organic acids. During the infection process calcium concentrations are expected to increase by cell wall lytic activities of the rapid growing hyphae and by autolysis of host cells. The vesicles with calcium precipitates that we found in actively growing hypha might represent transport vehicles for removing calcium from the infection front and translocate it to the older part of hyphae in the already destroyed host tissue where it is deposited as calcium oxalate crystals. Normally, in mycorrhizal fungi or fungi growing on litter oxalate crystals develop close to the hyphal tip [17]. Wood rotting fungi use oxalic acid not only for the degradation of lignocellulose, but also for detoxification of copper compounds and at the same time permits detoxification of calcium in the environment [16]. This kind of detoxification mechanism using oxalic acid might work as well as in pathogenesis of *S. sclerotiorum*. In this sense oxalic acid could be a key factor for the advanced and late infection stage. It balances the nutritional base to enable further hyphal development in the necrotic host tissue.

**Figure 12:**
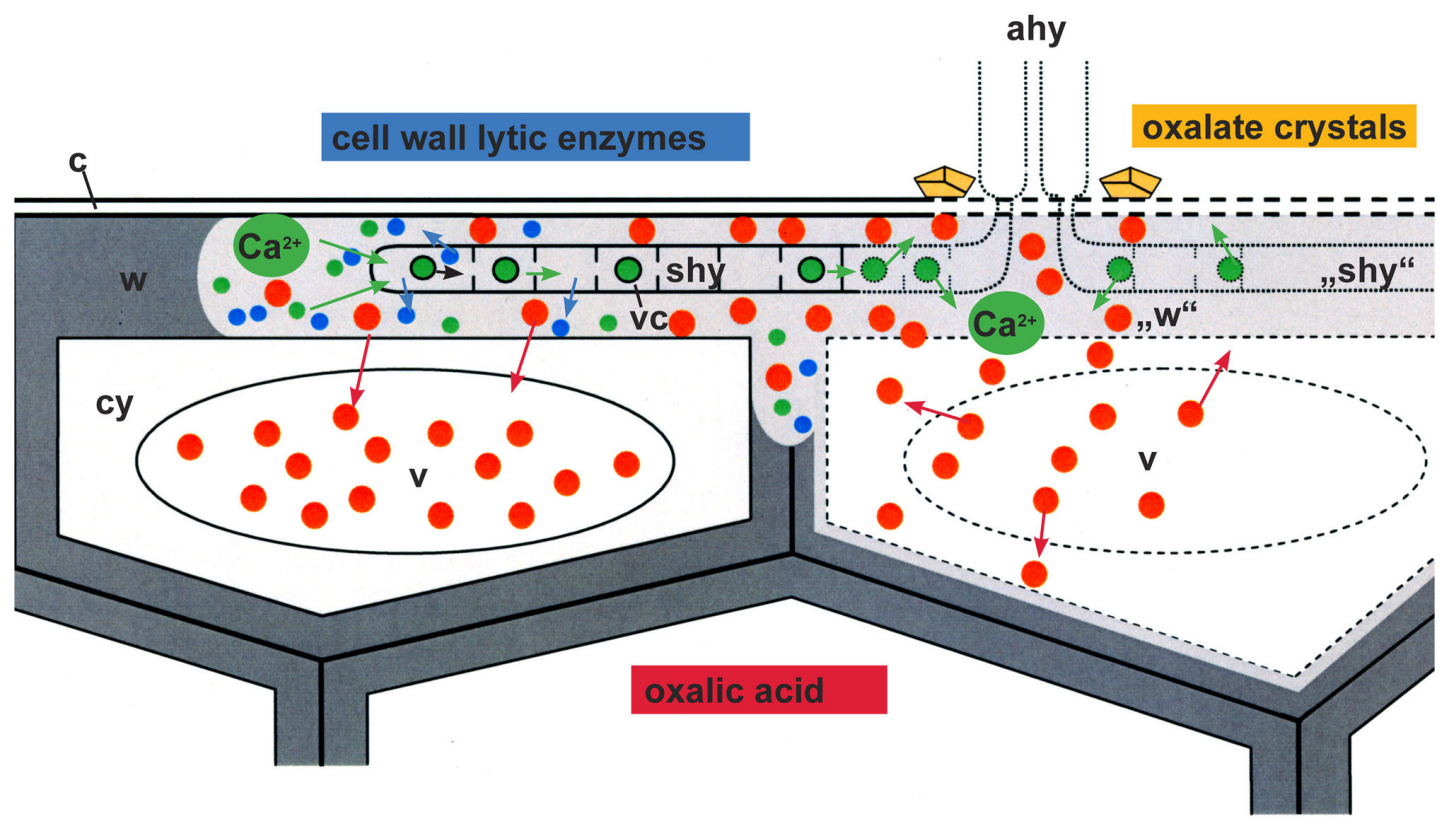
Infection model illustrating the complex interaction between *S. sclerotiorum* and its host cells sunflower. Scheme of two epidermal cells infected by hyphae of *S. sclerotiorum*. After penetration of the cuticle (c) by appressorial hyphae (ahy), subcuticular hyphae (shy) grow in the outer epidermal wall layer (w) and live on the cell wall matrix (intact wall in dark grey, degraded cell wall matrix in light grey (“w”). Subcuticular hyphae (shy) secrete cell wall lytic enzymes (blue dots) and oxalic acid (red dots) that do not kill the host cells immediately (intact cell compartments in continuous lines; degraded cell compartments in broken lines). While the enzymes degrade the matrix of the host cell walls and set free calcium (green dots), oxalic acid (red dots) is metabolized by the host cells and stored in the host cell vacuoles (v). Calcium is taken up by the growing hyphae in vesicles (vc) and translocated back to the older part (“shy”) to prevent un-physiological high calcium concentrations. Finally, host cells die (broken lines), because the degradation of cell walls advances and the concentration of oxalic acid rise. Oxalic acid (red dots) and also vacuolar enzymes of the host cells are set free and an autolytic degradation start from the inner side of the host cell walls contributing to the development of necrotic tissue. After death of the senescent hyphae (broken lines), calcium is again set free and is reacting with oxalic acid building stable calcium oxalate crystals (yellow) in the necrotic tissue around the functionless appressorial hyphae (ahy) of the infection cushion.

As a conclusion of reported and our current observations, we propose a model for the interaction between *S. sclerotiorum* and its host cells where oxalic acid plays an additional new role during the infection process (Figure 12). After penetration, subcuticular hyphae develop in the outer epidermal layer and live on the cell wall matrix. They secrete cell wall lytic enzymes and eventually oxalic acid that do not kill the host cells immediately. While the enzymes degrade the matrix of the host cell walls and release calcium, oxalic acid is metabolized by the host cells and stored in their vacuoles. Calcium is taken up by the growing hyphae in vesicles and translocated back to the older hyphal part to prevent un-physiological high concentrations at the infection front. Eventually, host cells die because the degradation of cell walls progress and the concentration of oxalic acid rise. Vacuolar oxalic acid and enzymes of the host cells are released and an autolytic degradation starts from the inner, cytoplasmic side of the host cell walls contributing to the development of lesions. After death of hyphae, calcium is again released and is reacting with oxalic acid building stable calcium oxalate crystals in the necrotic tissue.

## Materials and Methods

### Plant and fungal material

All investigations were carried out with the inbred line A89 of *Helianthus annuus* L, susceptible to *S. sclerotiorum*, kindly provided by V. Hahn, Landessaatzuchtanstalt, Universität Hohenheim. Plants were grown in soil for 4-8 weeks in a growth chamber at 16° C, 80% humidity, 16 hrs daylight, without additional fertilisation.

*Sclerotinia sclerotiorum* isolate ‘Rostock’ was obtained from H. Buchenauer, Institut für Phytomedizin, Universität Hohenheim, and maintained as sclerotia from diseased sunflower plants. For inoculum production, sclerotia were surface sterilized in sodium hypochlorite for 5 min, washed for 1 h in sterile, distilled water and cut in half. The halves were placed on potato dextrose agar (PDA) and kept at room temperature (RT) until the plate was densely covered by mycelium. Then a mycelium plug was transferred to malt agar and incubated at RT for 3 days.

### Inoculation method of *S. sclerotiorum* on sunflower hypocotyl

Pieces of sunflower hypocotyl about 5 cm in length, 4, 5 or 6-weeks old, were surface-sterilized in 1% sodium hypochlorite for 2 min, thoroughly washed with sterile water and placed on water agar in 9 cm Petri dishes. Agar plugs about 3 mm in diameter of 3 days old mycelium of *S. sclerotiorum* grown on malt agar were placed in a distance of about 1 mm to the hypocotyl, so that it was possible to study the attachment of hyphae, the development of the infection cushions, and the progressive infection process by using a stereomicroscope (Figure 1). Petri dishes were kept in a growth chamber at 16° C with a photoperiod of 16 h. Samples were taken for light microscopy (LM), conventional scanning electron microscopy (SEM) and low temperature scanning electron microscopy (LTSEM), and also for transmission electron microscopy (TEM), 12-24 hours post inoculation (hpi), early infection stage, 36-48 hpi, advanced infection stage, and 72 hrs hpi, late infection stage. Samples of non-infected hypocotyl treated in the same way, but without mycelium covered agar plugs, were used as control.

### Investigation of the infection process in fresh epidermal strips by light microscopy (LM) and histochemical staining of calcium oxalate

Fresh epidermal strips were removed from sunflower hypocotyl using a razor blade and fine forceps. They were stained with the protein-specific dye Coomassie blue according to the method of Wolf and Fric [46] to follow the fungus growing behaviour on the epidermal layer.

Histochemical identification of calcium oxalate in light microscopy was performed according to Yasue [19]. Infected and non-infected epidermal strips were soaked in 5% acetic acid to remove CaCO_3_ and Ca_3_(PO_4_)_2_. After washing in double distilled water, they were kept in 5% AgNO_3_ for 15 min in a dark chamber and washed again in double distilled water. The final dark brown staining of the calcium oxalate was achieved with saturated rubeanic acid in 70% ethanol for 1 min. After washing in 50% ethanol and in double distilled water, samples were kept in a mixture of acetic acid/glycerol/distilled water (5:20:75) until used for microscopy.

All samples were examined in bright field microscopy using an Axioplan light microscope (Zeiss, Göttingen, Germany) coupled to a 35 mm camera or a digital camera (Canon 95A).

### Investigation of the infection process by conventional scanning electron microscopy (SEM) and low temperature scanning electron microscopy (LTSEM)

For conventional SEM, 1-2 cm pieces of hypocotyl carrying infection sites were fixed in 4% glutaraldehyde in 0.1 M cacodylate buffer (pH 7,2) for 20 h, postfixed in buffered 1% osmium tetroxide for 20 h, and washed three times in distilled water. After dehydration in a series of acetone (30, 50, 75, 100, 100%), samples were critical point dried (CPD 020, Balzers, Union), mounted on Al-stubs and sputtered with gold–palladium (SCD 040, Balzers, Union).

Before the first fixation step, some samples in the advanced and late state of infection (48 hpi, 72 hpi) were covered with a mixture of albumin/glycerol to prevent loss of tissue during preparation. After fixation, dehydration and critical point drying (details see above) samples were quickly frozen in liquid nitrogen and broken. The infection sites on the surface and the fraction face of hypocotyls were sputtered with gold–palladium and investigated in a scanning electron microscope DSM 940 (Zeiss) at 5-7 kV.

Low temperature scanning electron microscopy (LTSEM) of infection stages were performed in the lab of R. Guggenheim at Basel University to follow exudation around hyphae and calcium oxalate crystal development. Infected hypocotyls of about 0.5–1 cm in length were mounted on specimen holder using low-temperature mounting medium (TBS, EMS, Washington, U.S.A.) and rapidly frozen in liquid nitrogen. Frozen samples were transferred to a Balzers cryopreparation unit SCU 020 attached to a JEOL JSM 6300 scanning electron microscope and sputter-coated with gold. The samples were investigated at a stage temperature of -165° C, using an accelerating voltage between 5 k and 25kV.

### Investigation of the infection process by light- and transmission electron microscopy (TEM)

For transmission electron microscopy small samples of tissue around infection cushions 0.5 mm x 0.5 mm x 2.0 mm in size were cut with a razor blade and immediately fixed in buffered 2.5% glutaraldehyde (0.1 M sodium cacodylate buffer, pH 7.2) for 1 h. After washing in buffer, a second fixation step in buffered 1% osmium tetroxide for 1 h followed. Dehydration was performed in a series (30, 50, 75, 100, 100%) of ethanol when using LR-White or acetone when using Epon. Samples were embedded in LR-White (Science Service, München, Germany) or Epon (Plano, Wetzlar, Germany). Series of semi-thin sections (1 µm) of infected tissue of different areas (hyphal tips of subcuticular hyphae, middle parts, and directly around infection cushions) were produced, stained with toluidine blue (0.5% aqueous solution), and investigated by light microscopy (Axioplan, Zeiss, Germany) before ultrathin sectioning. Series of ultrathin sections were prepared using an Ultracut UCT microtome (Leica, Wetzlar, Germany). The sections were transferred to Pioloform and carbon coated copper grids, stained with uranylacetate and lead citrate, and examined at 60 kV in an EM 10 transmission electron microscope (Zeiss, Oberkochen, Germany).

### Investigation of infection sites by TEM after precipitation of calcium by potassium pyroantimonate

The potassium pyroantimonate fixation method is a useful technique to localize calcium in cell compartments [47,48]. Samples of infection sites (1.0 mm x 1.0 mm x 0.5 mm in size) of four weeks old sunflower hypocotyls infected by *S. sclerotiorum* were prepared 24 hpi and 72 hpi for TEM at RT. They were fixed in 2.5% glutaraldehyde buffered with 0.1 M potassium phosphate buffer pH 7,6 for 2 h. A second fixation step in buffered (same buffer as described above) 2.5% glutaraldehyde with 1% potassium pyroantimonate for 4 h and three steps in buffered 1% potassium pyroantimoniate (20 min each) followed. After 14 h in buffered 1% osmium tetroxide with 1% potassium pyroantimonate samples were washed three times for 20 min in double distilled water and dehydrated in a series of ethanol (30, 50, 75, 95, 100, 100%. They were embedded in LR-White (Science Service, München) and polymerized at 60° C for 24 h. Ultrathin sectioning and investigation was the same as described above.

### The effect of oxalic acid on living, non-infected sunflower hypocotyls

Young (8 days old) and fully developed hypocotyls (6 weeks old) were used to investigate the effect of oxalic acid on non-infected living tissue of sunflower hypocotyls. Drops of 1 µl (8 days old hypocotyls) or 5 µl (6 weeks old hypocotyls) of oxalic acid (5 mM) were applied on the surface of hypocotyls. The epidermal layers were perforated with a fine needle to allow the oxalic acid to penetrate for 30 min. Controls were treated the same way but with drops of tap water. Samples of the treated tissue were taken and prepared for conventional TEM (see above).

For precipitating oxalate, some samples were treated with 10 mM CaCl_2_ in 2.5% glutaraldehyde for 1 h after the first fixation step with 1 h in 2.5% buffered glutaraldehyde. The first fixation step and the following preparation steps were the same as described above for TEM.

### Effect of oxalic acid on inactivated, non-infected sunflower hypocotyls

After the puzzling results of the effect of oxalic acid on non-infected tissue, that indicated that the host cells were able to reduce the fungal oxalic acid so that no oxalate crystals developed after application of CaCl_2_ the effect of oxalic acid was also tested on inactivated host tissue. Samples (1mm x1mm x 0.5mm in size) of four week old non-infected hypocotyls were cut with a razor blade and immediately fixed 2,5% buffered glutaraldehyde for 1 h to stop all biochemical activities. Then samples were exposed to 5 mM oxalic acid in fixation solution (see above) for 30 min. After a short washing step in distilled water, a second fixation step followed in 2.5% glutaraldehyde in aqueous 10 mM CaCl_2_ to precipitate oxalate. The following preparation steps were the same as described for conventional TEM. In control samples the oxalic acid treatment was omitted.
